# Complement Plays a Critical Role in Inflammation-Induced Immunoprophylaxis Failure in Mice

**DOI:** 10.3389/fimmu.2021.704072

**Published:** 2021-06-25

**Authors:** Vicente Escamilla-Rivera, Manjula Santhanakrishnan, Jingchun Liu, David R. Gibb, James E. Forsmo, Ellen F. Foxman, Stephanie C. Eisenbarth, C. John Luckey, James C. Zimring, Krystalyn E. Hudson, Sean R. Stowell, Jeanne E. Hendrickson

**Affiliations:** ^1^ Department of Laboratory Medicine, Yale University School of Medicine, New Haven, CT, United States; ^2^ Department of Pathology & Laboratory Medicine, Cedars-Sinai Medical Center, Los Angeles, CA, United States; ^3^ Department of Biomedical Engineering, Georgia Institute of Technology, Atlanta, GA, United States; ^4^ Department of Immunobiology, Yale University School of Medicine, New Haven, CT, United States; ^5^ Department of Pathology, University of Virginia, Charlottesville, VA, United States; ^6^ Department of Pathology and Cell Biology, Columbia University Irving Medical Center, New York, NY, United States; ^7^ Department of Pathology, Brigham and Women’s Hospital, Joint Program in Transfusion Medicine, Harvard Medical School, Boston, MA, United States; ^8^ Beth Israel Deaconess Medical Center, Harvard Glycomics Center, Harvard Medical School, Boston, MA, United States; ^9^ Department of Pediatrics, Yale University School of Medicine, New Haven, CT, United States

**Keywords:** red blood cell, complement, antibody, transfusion, alloimmune

## Abstract

Complement impacts innate and adaptive immunity. Using a model in which the human KEL glycoprotein is expressed on murine red blood cells (RBCs), we have shown that polyclonal immunoprophylaxis (KELIg) prevents alloimmunization to transfused RBCs when a recipient is in their baseline state of heath but with immunoprophylaxis failure occurring in the presence of a viral-like stimulus. As complement can be detected on antibody coated KEL RBCs following transfusion, we hypothesized that recipient complement synergizes with viral-like inflammation to reduce immunoprophylaxis efficacy. Indeed, we found recipient C3 and C1q were critical to immunoprophylaxis failure in the setting of a viral-like stimulus, with no anti-KEL IgG alloantibodies generated in C3^-/-^ or C1q^-/-^ mice following KELIg treatment and KEL RBC transfusion. Differences in RBC uptake were noted in mice lacking C3, with lower consumption by splenic and peripheral blood inflammatory monocytes. Finally, no alloantibodies were detected in the setting of a viral-like stimulus following KELIg treatment and KEL RBC transfusion in mice lacking complement receptors (CR1/2^-/-^), narrowing key cells for immunoprophylaxis failure to those expressing these complement receptors. *In-vitro* studies showed complement fixed opsonized RBCs were significantly less likely to bind to B-cells from CR1/2^-/-^ than wild type mice, potentially implicating lowered B-cell activation threshold in the presence of complement as being responsible for these findings. We thus propose a two-hit model for inflammation-induced immunoprophylaxis failure, where the first “hit” is recipient inflammation and the second “hit” is complement production/sensing. These results may have translational relevance to antigen-antibody interactions in humans.

## Introduction

Alloimmunization to red blood cells (RBCs) can be significant in transfusion, transplantation, and pregnancy settings. Polyclonal anti-D (RhIg) immunoprophylaxis has been used for over half a century as an effective preventive measure for alloimmunization to RBCs expressing the RhD antigen in pregnancy (1). Immune regulation by antibody-antigen interactions is well known in different immunological settings; however, the mechanism(s) by which RhIg works remains controversial. Further, it is not understood why breakthrough anti-D cases occur even when RhIg is administered properly.

We have previously described a murine model in which polyclonal anti-KEL (KELIg) immunoprophylaxis prevents alloimmunization to transfused RBCs expressing the human KEL glycoprotein when administered to recipient mice in their baseline state of heath (2). However, immunoprophylaxis fails in the setting of viral-like inflammation with poly (I:C) (3). We further showed that although type 1 interferon (IFNα/β) generation and sensing is sufficient to lead to immunoprophylaxis failure, it is not required (3). As such, the mechanism(s) leading to this breakthrough alloimmunization are only partially understood.

Complement plays a fundamental role in regulating immune responses, and we have previously reported that anti-KEL antibodies activate complement upon binding to KEL RBCs. Accordingly, we hypothesized that complement activation is involved in immunoprophylaxis failure and here we report that complement is required for immunoprophylaxis failure in the setting of poly (I:C). Complement receptors are likewise required, supporting a model of C3 opsonization of antibody bound RBCs that directly impacts immunoprophylaxis and its failure. These findings elucidate new and undescribed mechanistic details of antigen-antibody interactions.

## Methods

### Mice

C57BL/6NCrl (B6) mice (strain code 027) were purchased from Charles River (Wilmington, MA). Complement C3 knockout, B6;129S4-C3/J(stock#003641) (C3^-/-^), C1qa knockout, B6(Cg)-C1qatm1d(EUCOMM)Wtsi/TennJ (stock #: 031675 (C1q^-/-^), and CR1/2 knockout, B6.129S7(NOD)-Cr2tm1Hmo/J (Stock #008225) (CR1/2^-/-^) mice were purchased from Jackson (Bar Harbor, ME). Transgenic mice expressing the entire human KEL glycoprotein were previously generated; the mice used for these experiments have been previously described as “KEL2B” and express the KEL2 antigen in addition to the Js^b^ antigen, the Kp^b^ antigen, and other antigens in the KEL family on their RBCs (4). In this study they are referred to as “KEL” mice as the protein being studied includes the entire human KEL glycoprotein. All mice were housed in Yale University’s animal facilities. All mice were 8-12 weeks of age and had a history of being backcrossed to the C57BL/6 background for at least 8 generations. All procedures and protocols were approved by Yale University’s Institutional Care and Use Committee.

### KELIg Generation, Immunization, and Other Mouse Treatments

Polyclonal KELIg antisera was generated as previously described (2), by transfusing transgenic murine KEL RBCs into B6 recipients pre-treated with an intraperitoneal injection of 100 μg of high molecular weight poly (I:C) (Invivogen, San Diego, CA) a total of 3 times, separated by two weeks between each transfusion. Pooled sera collected 2-4 weeks after the final transfusion was tested for KEL binding ability by flow crossmatch with KEL or control B6 RBCs as targets; all IgG subtypes are represented (2). Following dose titration experiments, recipient mice were passively transferred with enough KELIg to lead to maximal RBC clearance (approximately 15 µL per experiment). In some experiments, poly (I:C) was administered approximately 3 hours prior to RBC transfusion.

### Blood Collection, Labeling, and Transfusion

Donor KEL or wild type B6 RBCs were collected into anticoagulant preservative solution (CPDA, citrate phosphorus dextrose adenine, Jorgensen Labs, Henry Schein, Melville, NY), leukoreduced over a syringe filter (Pall Corporation, Port Washington, NY), and washed to remove residual citrate. Prior to transfusion in some experiments, B6 RBCs were labeled with chloromethylbenzamido 1,1’-dioctadecyl-3,3,3’,3’-tetramethylindocarbocyanine perchlorate (CM-DiI) and KEL RBCs were labeled with 3,3’-dihexadecyloxacarbocyanine perchlorate (DiO) according to the manufacturer’s instructions (Molecular Probes, Eugene, OR) and as previously described (5). Recipient mice were transfused *via* lateral tail with 50 µL of KEL RBCs (in addition to control wild type RBCs in RBC recovery experiments). Survival of the transfused RBCs was determined by calculating the ratio of circulating DiO to DiI RBCs in recipients at select time points post-transfusion.

### Flow Cytometry

Sera was collected at multiple time points and anti-KEL IgG responses were measured using a flow cytometric crossmatch assay with antigen positive (KEL) or antigen negative (B6) RBC targets. The secondary antibody was goat anti-mouse IgG (Jackson Immunoresearch, West Grove, PA). The antigen specific response [adjusted mean fluorescence intensity (MFI)] was determined by subtracting the signal of serum with antigen negative B6 RBCs from that of serum with antigen positive RBCs. In experiments involving KELIg, the D0 timepoint was normalized to a fluorescence intensity of 1000 and a similar normalization was completed for the other timepoints. Transfused RBCs were analyzed for the KEL antigen by incubating with KELIg followed by anti-mouse IgG.

Following transfusion of KEL RBCs, complement was evaluated on the visualized RBC surface using antibodies against C3 (Cl7503B, Cedarlane, Ontario, Canada), C4 (clone 16D2, Santa Cruz), or Factor B (CL8824AP, Cedarlane, Ontario, Canada) followed by streptavidin or a fluorescently conjugated donkey anti-rabbit antibody (BioLegend, San Diego, California).

In other experiments, splenic cell subsets were evaluated following the transfusion of labeled RBCs. Spleens were harvested into ice-cooled RPMI 1640 media, finely minced with a razorblade, and processed into single-cell suspensions by passing the samples through a 70 µm nylon cell strainer and collecting them on ice-cold FACS buffer (DPBS Modified, 0.2% BSA, 0.5 M EDTA). RBCs were lysed with ammonium chloride and splenocytes were treated with Fc block (anti-mouse CD16/CD32; BD Biosciences, San Jose, CA) followed by incubation with cell surface antibodies. Antibodies against CD19 (clone 6D5), TCRβ (clone H57-597), CD11b (clone M1/70), CD11c (clone N418), CD8a (clone 53-6.7), F4/80 (clone BM8), CD115 (CSF-1R, clone AFS98), Ly-6G/Ly-6C (GR-1, clone RB6-8C5), and PDCA-1 (CD137, BST2, clone 927) were purchased from BioLegend (San Diego, CA). Anti-TER119 (clone TER119) was purchased from Invitrogen (Thermo Fisher Scientific Carlsbad, CA). Zombie Violet viability dye was purchased from BioLegend. For the *in-vitro* experiments involving peripheral blood WBCs, antibodies used also included CD19 (clone 6D5), B220 (clone RA3-6B2), CD21/35(clone 7E9), and CD23 (clone B3B4). Samples were analyzed on a BD LSR II cytometer, BD FACSCalibur, Miltenyi MACSQuant, or Beckman coulter CytoFlex S.

### *In-Vitro* Experiments

*In-vitro* RBC experiments were completed by incubating KEL RBCs with KELIg, followed by the addition of serum, serum treated with EDTA, or serum treated with EGTA-Mg (Millipore Sigma); the serum was freshly collected from wild type mice and kept on ice until the addition of KEL RBCs. KELIg was diluted in GVB buffer with Mg and Ca (Complement Technology) and the cells were washed in GVB buffer without Ca or Mg. Other *in-vitro* experiments involved incubating KEL RBCs (or wild type RBCs) with or without KELIg, following by incubation with sera or no sera, followed by incubation with peripheral blood WBCs from wild type mice or CR1/2^-/-^ mice, followed by staining for flow cytometric evaluation.

### Statistics and Images

All statistical analysis was performed using Graph Pad Prism software (San Diego, CA). A Mann Whitney U test was used to determine significant differences between two groups, and ANOVA with Tukey’s multiple comparisons test was completed in relevant experiments. Error bars represent one standard deviation, and significance was determined by a p-value less than 0.05. The visual abstract was created using BioRender.com.

### Data Sharing Statement

For original data, please contact jeanne.hendrickson@yale.edu.

## Results

### Complement Is Fixed on KEL RBCs *In-Vivo* and *In-Vitro* in Response to Polyclonal Anti-KEL (KELIg)

**Figure 1:**
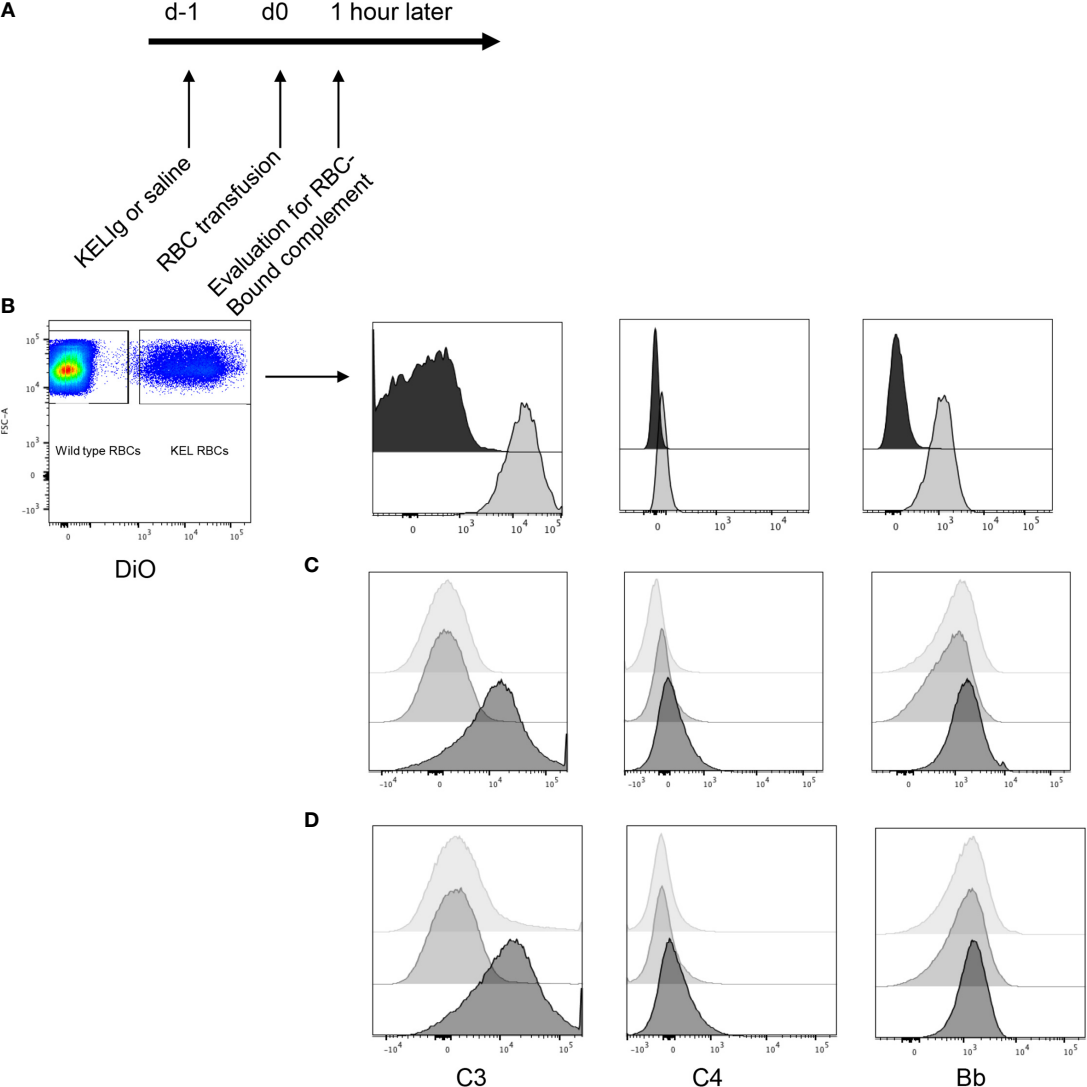
Complement is fixed on KEL RBCs *in-vivo* and *in-vitro* in response to polyclonal anti-KEL (KELIg). **(A)** General *in-vivo* experimental design: recipients were treated with or without KELIg and transfused with DiO labeled KEL RBCs. **(B)** C3, C4, and Factor B were measured on recovered DiO positive KEL RBCs 1-hour post-transfusion; black filled histogram shows condition without KELIg; grey shaded histogram shows condition with KELIg. **(C)** *In-vitro* experiments were completed with KELIg incubated with KEL RBCs in the presence (darkest histogram) or absence (lightest histogram) of serum; an additional condition included saline incubated with KEL RBCs in the presence of serum (medium grey histogram). **(D)** Additional *in-vitro* conditions included KELIg incubated with KEL RBCs in the presence of serum (darkest histogram), in the presence of serum with EGTA-Mg (lightest histogram) or serum with EDTA (medium grey histogram). These data are representative of 3 independent experiments; p < 0.05 for C3 detection on RBCs in the presence or absence of KELIg.

To evaluate the role of complement in immunoprophylaxis failure, we first tested whether passively transferred anti-KEL IgG (KELIg) leads to fixation by different complement components. Following KELIg infusion, KEL RBCs labeled with a lipophilic dye were transfused and RBCs recovered one hour later were evaluated for bound complement proteins (**Figure 1A** shows the experimental design). Complement C3, C4, and Factor Bb were detected bound to the recovered DiO labeled KEL RBCs in mice treated with KELIg, but not in control mice treated with saline (**Figure 1B**).

To determine which complement pathway was responsible, *in-vitro* experiments were completed. KEL RBCs were incubated with or without KELIg. After washing away unbound KELIg, KEL RBCs were incubated with serum from wild type mice and RBC-bound complement C3, C4, and Factor Bb were measured. Similar to results from *in-vivo* experiments, C3 was readily detected bound to KEL RBCs in the condition involving KELIg and serum; C4 and Bb were more difficult to detect (**Figure 1C**). No complement was detected in KEL RBCs with serum or KEL RBCs with KELIg without serum control samples (**Figure 1C**). Other conditions included serum treated with EDTA (which blocks complement) or serum treated with EGTA and magnesium (which blocks the classical pathway of complement). Very little C3 could be detected under either of these conditions (**Figure 1D**), indicating the classical (antibody-mediated) complement pathway is most likely responsible for the RBC bound complement.

### Recipient Complement Is Required for KELIg Immunoprophylaxis Failure in Mice Treated With Poly (I:C)

**Figure 2:**
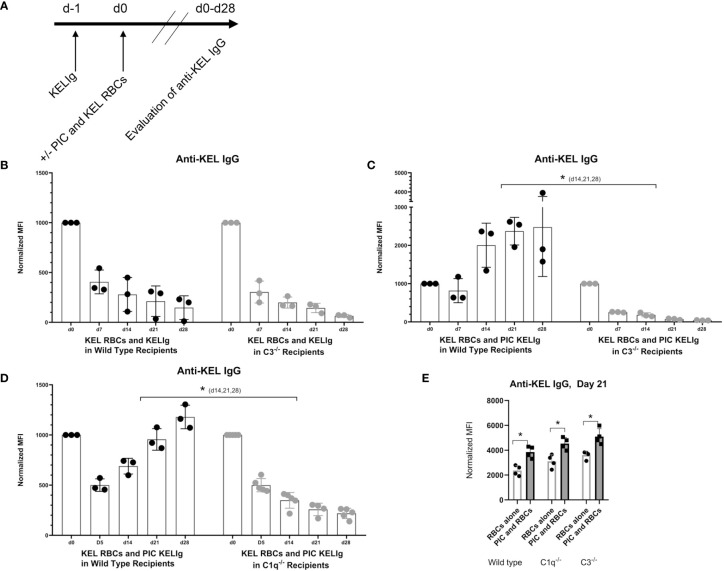
Recipient C3 and C1q contribute to KELIg immunoprophylaxis failure in the setting of poly (I:C). **(A)** General *in-vivo* experimental design: recipients were infused with KELIg and transfused the following day in the absence or presence of poly (I:C) treatment, with anti-KEL responses evaluated longitudinally. **(B)** Anti-KEL IgG responses in wild type compared with C3^-/-^ recipients transfused in the absence of poly (I:C). **(C)** Anti-KEL IgG responses in wild type compared with C3^-/-^ recipients transfused in the presence of poly (I:C). **(D)** Anti-KEL IgG responses in wild type compared with C1q^-/-^ recipients transfused in the presence of poly (I:C). **(E)** Baseline impact of recipient treatment with poly (I:C) on alloimmune responses to KEL RBCs in the absence of KELIg. These data are representative of 2-3 independent experiments, with 3-5 mice/group/experiment; error bars indicate standard deviation between individual mice. *p < 0.05 **(C, D)** d14,21,28, and all comparisons for **(E)**.

Since we observed that KELIg fixes complement (**Figure 1**), we tested the role of complement in the efficacy of immunoprophylaxis for recipients in their baseline state of health and following inflammation induced by poly (I:C). We found that immunoprophylaxis efficacy of KELIg when recipients are in their baseline state of health is not dependent on complement fixation, as KELIg remained as effective at preventing active anti-KEL formation in transfusion recipients lacking C3 as in wild type recipients (6) (**Figures 2A, B**). As shown in prior studies (2), the day 0 time point in **Figure 2B** and other figures involving KELIg is a measure of passive anti-KEL detected following KELIg administration; the day 14 time point represents a mixture of passively administered KELIg and actively formed anti-KEL, and by day 28 much of the passively administered KELIg is gone. However, in contrast to what we observed in wild type mice, poly (I:C) administration to C3^-/-^ recipients did *not* lead to immunoprophylaxis failure (**Figure 2C**). This failure was likewise prevented in mice lacking C1q, which is upstream of C3 in the classical pathway of complement activation (**Figure 2D**) – suggesting that the classical pathway is required. Loss of poly (I:C) immunoprophylaxis failure was not due to an elimination of general poly (I:C) responsiveness in C3^-/-^ or C1q^-/-^ mice, as poly (I:C) induced a significant increase of anti-KEL IgG in response to KEL RBC transfusion in both strains (in the absence of pretreatment with KELIg) (**Figure 2E**).

### Recipient Complement Does Not Significantly Impact KEL RBC Clearance After KELIg in the Presence of Poly (I:C)

**Figure 3:**
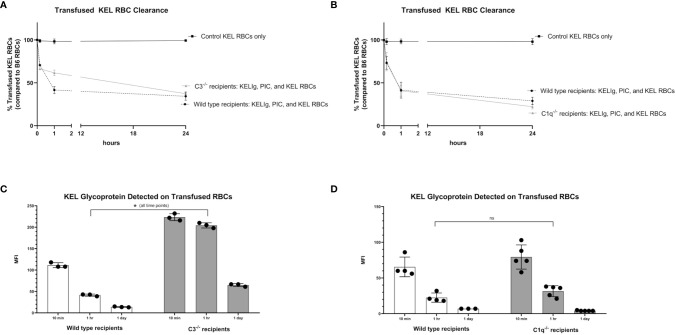
Recipient complement, KEL RBC clearance, and KEL antigen expression. KEL RBCs were labeled with DiO and mixed with wild type RBCs labeled with DiI; this mixture was transfused into recipients that had been infused with KELIg and treated with poly (I:C). **(A)** shows wild type compared with C3^-/-^ recipients; **(B)** shows wild type compared with C1q^-/-^ recipients. Recovered DiO labeled RBCs were then evaluated for KEL glycoprotein expression by flow cytometry, after incubation with KELIg and fluorescently conjugated anti-mouse IgG; **(C)** shows wild type recipients compared with C3^-/-^ recipients; **(D)** shows wild type recipients compared with C1q^-/-^ recipients. These data are representative of 2-3 independent experiments with 3 mice/group/experiment; error bars indicate standard deviation between mice. *p < 0.05 for all comparisons in **(C)** and p = ns, not significant for all comparisons in **(D)**.

Given that immunoprophylaxis failure occurred in wild type mice but not in C3^-/-^ or C1q^-/-^ mice, we investigated the role of complement in clearance rates of transfused KEL RBCs. Following passive administration of KELIg and treatment with poly (I:C), recipient mice (wild type, C3^-/-^, and C1q^-/-^) were transfused with DiO labeled KEL RBCs mixed with DiI labeled wild type RBCs. Blood was taken from recipient mice at 10 minutes, 1 hour, and 24 hours post-transfusion and the ratio of DiO KEL RBCs to DiI wild type RBCs was evaluated. As we have observed when KELIg is administered in the absence of inflammation (6), C3^-/-^ mice had slightly delayed RBC clearance compared with wild type mice in the presence of inflammation (**Figure 3A**), with C1q^-/-^ mice having similar clearance patterns compared with wild type mice (**Figure 3B**); **Supplemental Figure 1** shows C3^-/-^ and C1q^-/-^ recipient RBC clearance following KELIg in the absence of poly (I:C).

The recovered DiO labeled KEL RBCs were then evaluated for the presence of the KEL glycoprotein antigen by incubating the recovered cells with anti-KEL antibody followed by fluorescently labeled mouse IgG. As previously shown, KEL antigen expression on RBCs remains stable at 10 minutes, 1 hour, and 24 hours post-transfusion in wild type mice transfused in the absence of KELIg (2), and antigen expression is similar in the presence or absence of poly (I:C) (3). The absence of C3 (**Figure 3C**), but not C1q (**Figure 3D**), delayed KEL glycoprotein antigen modulation compared with wild type mice after treatment with KELIg in the presence of poly (I:C), for reasons that are not entirely self-evident. Taken in combination, however, it is unlikely that either RBC clearance or antigen modulation are responsible for explaining the differences in immunoprophylaxis efficacy observed between complement deficient and wild type mice in the setting of inflammation.

### Recipient Complement Enhances KEL RBC Uptake by Inflammatory Monocytes After KELIg in the Presence of Poly (I:C)

**Figure 4:**
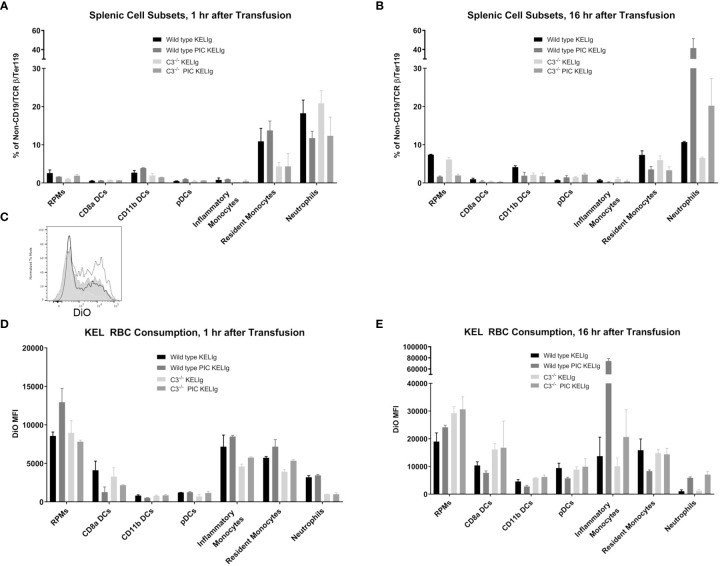
Poly (I:C) and KELIg immunoprophylaxis result in increased transfused KEL RBC consumption by splenic inflammatory monocytes in wild type compared with C3^-/-^ mice. DiO labeled KEL RBCs were transfused to wild type or C3^-/-^ mice treated with KELIg in the presence or absence of poly (I:C) and splenic cell subsets were evaluated at 1 and 16 hours post-transfusion **(A, B)**. **(C)** Representative histograms for DiO RBC fluorescence patterns of inflammatory monocytes at 16 hours post-transfusion, after first excluding TER119 positive RBCs on the exterior of the splenic cells; black open histogram is KELIg in wild type, dotted open histogram is KELIg and PIC in wild type; white shaded histogram is KELIg in C3^-/-^ and dotted shaded histogram is KELIg and PIC in C3^-/-^. DiO mean fluorescence intensity (MFI) of the splenic cell subsets was evaluated at 1 hour **(D)** and 16 hours **(E)** post-transfusion. These data are representative of 2-3 independent experiments with 3 mice/group/experiment; error bars indicate standard deviation between mice.

Complement is known to mediate antigen uptake by antigen presenting cells (APCs) (7). Thus, despite the relatively similar overall peripheral RBC clearance patterns in wild type mice compared with those lacking complement, we hypothesized that the incompatible KEL RBCs may be consumed by different APCs more likely to promote alloantibody formation or that the APCs consuming the transfused RBCs may be differentially activated in the presence of complement. To evaluate RBC consumption, wild type and C3^-/-^ mice were transfused with DiO labeled KEL RBCs and leukocyte subsets in their spleens were evaluated 1 hour and 16 hours later. First, we gated on single cells, live cells, and non-T/non-B/non-RBCs (**Supplemental Figure 2**). Next, splenic red pulp macrophages, dendritic cells (CD8α, CD11b and plasmacytoid), resident and inflammatory monocytes, and neutrophils were quantified by percentage one hour (**Figure 4A** **)** and 16 hours (**Figure 4B**) post-transfusion. Differences in these cell subsets were observed between genotypes as shown, with an increase in neutrophils after poly (I:C) treatment in wild type mice being particularly prominent by 16 hours post-transfusion. Next, RBC consumption of transfused DiO labeled KEL RBCs by each of these cell subsets was determined. One difference between wild type and C3^-/-^ mice following KELIg and poly (I:C), compared with KELIg alone, was the increase in DiO labeled KEL RBC consumption by inflammatory monocytes that occurred in wild type mice and that was seen to a lesser degree in C3^-/-^ mice (**Figure 4C** shows a histogram example at 16 hours post-transfusion); these changes were evident one hour post-transfusion (**Figure 4D**) but were even more pronounced by 16 hours post-transfusion (**Figure 4E**).

In addition, monocytes and neutrophils were evaluated in the peripheral blood at the 1 hour and 16-hour post-transfusion time points (**Supplemental Figures 3A, B**). Similar to trends observed in the spleen, peripheral blood inflammatory monocytes in the wild type mice had significantly more DiO signal inside them compared with those in the C3^-/-^ mice (**Supplemental Figures 3C, D**). Taken in combination, these findings suggest that in the setting of inflammation, complement fixation may direct the KEL antigen-containing RBCs towards antigen presenting cells more likely to ultimately contribute to a productive antibody response.

### Complement-Fixed KEL RBCs Bind *In-Vitro* to B-Cells From Wild Type Mice

**Figure 5:**
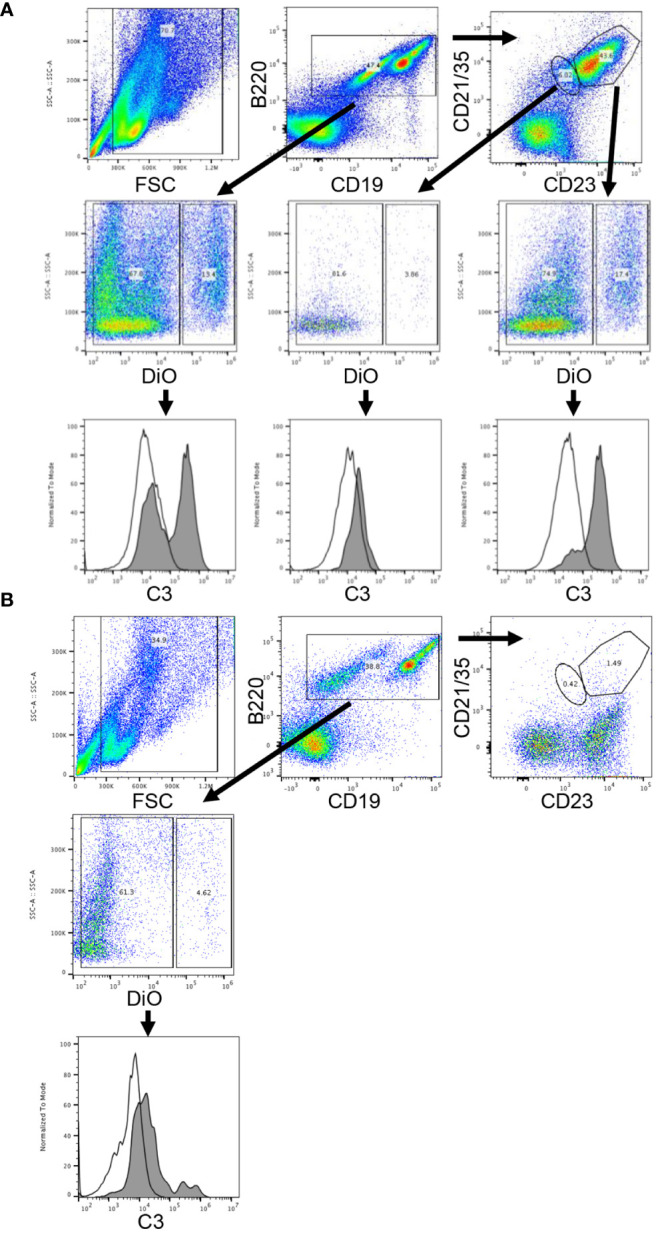
Complement-fixed KEL RBCs bind *in-vitro* to wild type donor derived B-cells but not to CR1/2^-/-^ B-cells. DiO labeled KEL RBCs were incubated with KELIg in the presence of sera, followed by incubation with peripheral blood derived WBCs from donor mice. **(A)** In wells using WBCs from wild type donor mice, CD19+B220+ B-cells cells were separated by DiO positivity and then by C3 positivity. The CD19+B220+ cells were next separated by their CD23 and CD21/35 expression, with the cells highest for CD23 and CD21/35 gated in the larger gate and those less strongly positive for CD23 and CD21/35 gated in the smaller gate; the DiO positive and negative populations were then evaluated for their C3 positivity. Shaded histograms are the DiO positive population, open histograms are the DiO negative population. **(B)** In wells using WBCs from CR1/2^-/-^ mice, CD19+B220+ B-cells cells were separated by DiO positivity and then by C3 positivity. Shaded histograms are the DiO positive population, open histograms are the DiO negative population. These data are representative of more than 3 independent experiments, with 2 involving CD23 and CD21/35 staining.

As the interaction of complement, antigen, and complement receptors has long been known to be critical for regulating antibody production (8), we next turned our attention to complement receptors on B-cells. Work by others has shown that complement receptors 1 and 2 (CR1/2) are present on follicular dendritic cells and B-cells in mice, and crosslinking of the B-cell receptor (BCR) and CR1/2 on B-cells lowers the activation threshold (9). As a first step to evaluate the role of complement binding to B-cells in our model, we incubated DiO labeled KEL or wild type RBCs with or without KELIg *in-vitro*. After washing away unbound KELIg, the RBCs were incubated with sera and white blood cells (WBCs). CD19+B220+ WBCs that were positive for DiO had a mixture of C3 positivity and C3 negativity under conditions including KEL RBCs + KELIg + sera, and CD19+B220+ WBCs that were negative for DiO were C3 negative (**Figure 5A**). Control conditions, including those lacking KEL RBCs, those lacking KELIg, and those lacking sera showed no C3 positivity (**Supplemental Figure 4**). To investigate which CD19+B220+ WBCs bound the complement fixed KEL RBCs, we utilized CD23 and CD21/35 staining. The CD19+B220+ cells most highly positive for CD23 and CD21/35 (largest gate) that were positive for DiO had uniform C3 positivity, whereas the CD19+B220+ cells with less strong CD23 and CD21/35 positivity (smallest gate) that were positive for DiO were predominantly C3 negative. The conditions were then expanded, comparing WBCs from CR1/2^-/-^ mice with those from wild type mice. The CD19+B220+ B-cells from CR1/2^-/-^ mice showed decreased binding to KEL RBCs compared with B-cells from wild type mice. Further, the CR1/2^-/-^ B-cells that bound DiO labeled KEL RBCs had less detectable C3 compared with those from wild type mice (**Figure 5B**). These results suggest that B-cells bind to opsonized, complement fixed KEL RBCs through CR1/2, raising a question of the role these receptors play in immunoprophylaxis failure.

### Complement Receptors (CR1/2) Are Necessary for KELIg Immunoprophylaxis Failure in the Presence of Poly (I:C)

**Figure 6:**
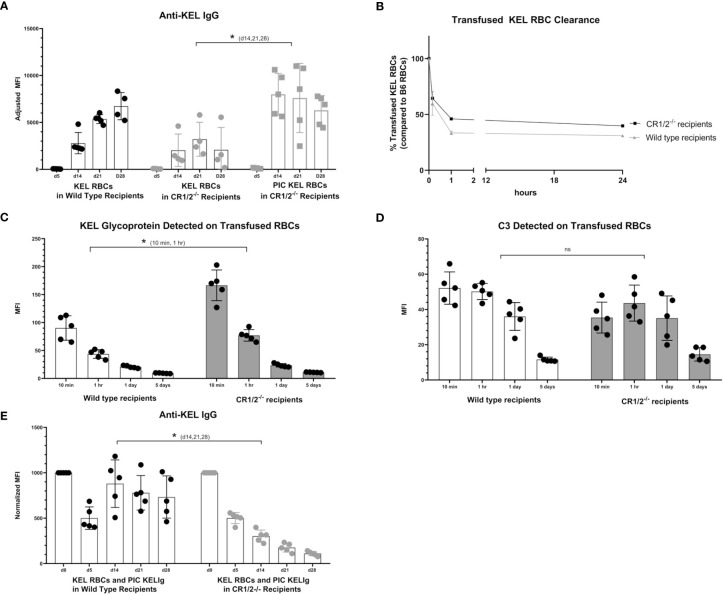
CR1/2 are required for KELIg immunoprophylaxis failure in the setting of poly (I:C). **(A)** KEL RBCs were transfused into wild type recipients or recipients lacking CR1/2, in the presence or absence of poly (I:C). **(B)** RBCs were labeled with DiO and mixed with wild type RBCs labeled with DiI; this mixture was transfused into wild type or CR1/2^-/-^ recipients that had been infused with KELIg and treated with poly (I:C). Recovered DiO positive KEL RBCs were evaluated for **(C)** KEL glycoprotein expression, and **(D)** bound complement C3. **(E)** Wild type or CR1/2^-/-^ recipients were infused with KELIg and transfused the following day in the absence or presence of poly (I:C), with anti-KEL responses evaluated longitudinally. *p < 0.05 for d14, 21 and 28 of **(A)** between CR1/2^-/-^ mice treated with or without poly (I:C); p < 0.05 for 10 min and 1-hour timepoints in **(C)**; p < 0.05 for d14, 21, and 28 in **(E)**. These data are representative of 2-3 independent experiments with 3 mice/group/experiment; error bars indicate standard deviation between mice. ns, not significant.

To evaluate the functional role of CR1/2 in the immunoprophylaxis setting of KELIg, we first needed to establish that these mice could generate an anti-KEL response following a KEL RBC transfusion. Similar to wild type mice, we observed that CR1/2^-/-^ recipients were able to generate anti-KEL IgG antibodies following a KEL RBC transfusion and to generate an enhanced response in the presence of poly (I:C), albeit lower in magnitude than that observed in wild type mice (**Figure 6A**). Next, we evaluated the response of CR1/2^-/-^ mice to KEL RBCs after prophylaxis with KELIg. As we have observed in wild type mice, KELIg prevented anti-KEL alloantibody responses in CR1/2^-/-^ mice (data not shown). Compared to wild type mice, the KEL RBCs recovered from the CR1/2^-/-^ mice had similar post-transfusion recover and survival as wild type recipients (**Figure 6B**), with delayed kinetics of KEL glycoprotein antigen modulation (**Figure 6C**) and a trend towards lower levels of bound C3 (**Figure 6D**). However, in the setting of poly (I:C)-induced inflammation and KELIg, immunoprophylaxis failure and breakthrough alloimmunization did not occur in CR1/2^-/-^ mice (**Figure 6E**). Taken together, these data indicate that CR1/2 are required for immunoprophylaxis failure and further emphasize the lack of an association between peripheral RBC clearance rates and outcome, supporting a model in which complement functions in breakthrough alloimmunization by promoting antibody-generating immune responses.

## Discussion

**Figure 7:**
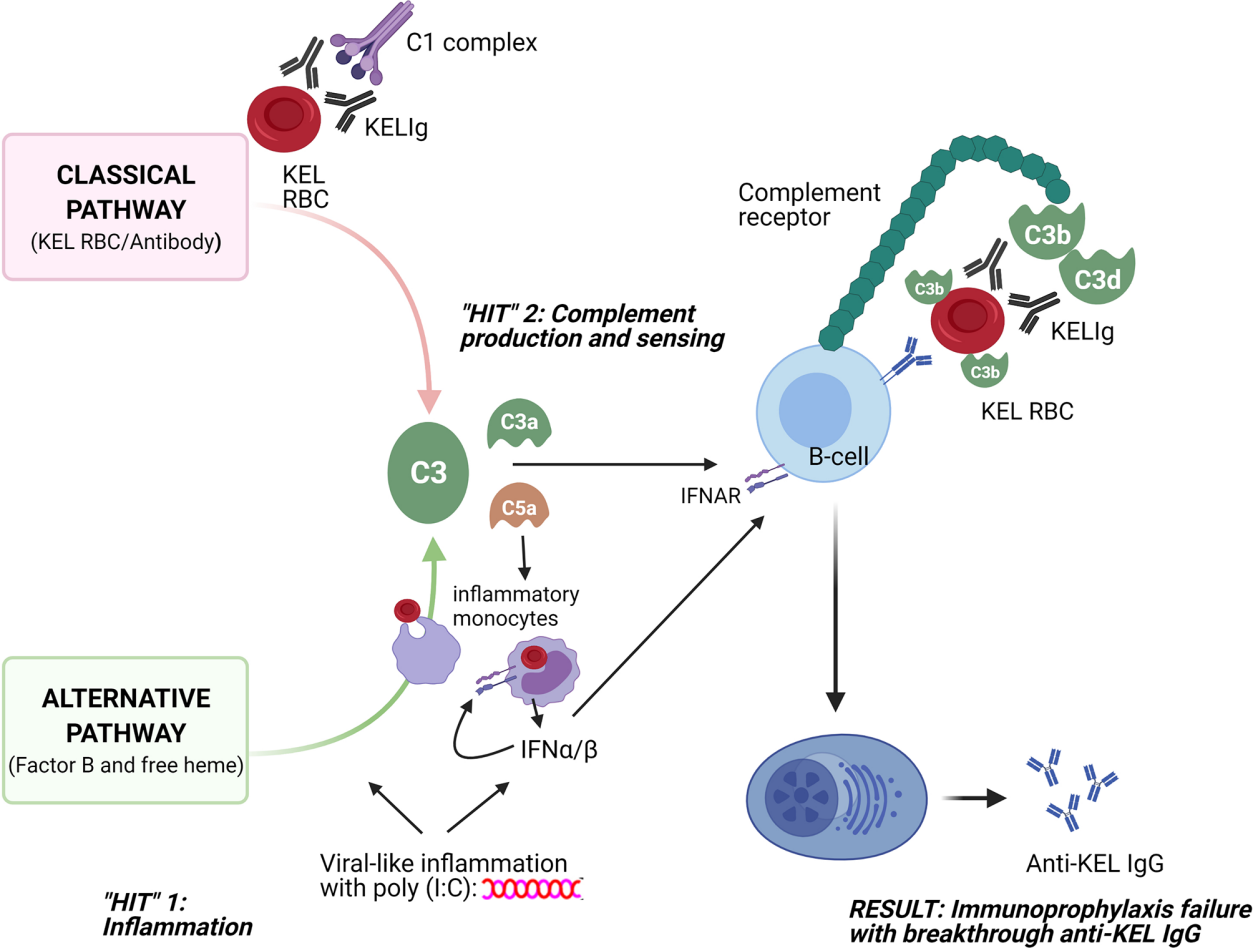
Schematic of 2 “hits” resulting in immunoprophylaxis failure in the setting of viral-like inflammation. The first “hit” involves recipient inflammation with poly (I:C), with resultant type 1 (α/β) interferon production among other things; the second “hit” involves complement generation through the classical pathway due to antibody/antigen interactions and through the alternative pathway due to poly (I:C) and potentially due to free heme generation. Consumption of opsonized RBCs by inflammatory monocytes likely also plays a key role, with type 1 IFN and C3a and C5a potentially increasing this consumption and with type 1 IFN generation likely having an impact on downstream B-cells. The sum of these two “hits” is that opsonized KEL RBCs with fixed complement bind to CR1/2 on B-cells, lowering the activation threshold for anti-KEL formation and resulting in inflammation-induced immunoprophylaxis failure.

These studies were undertaken to better understand why polyclonal anti-KEL IgG (KELIg) prevents alloimmunization when a recipient is exposed to KEL RBCs in a baseline state of health but fails to prevent alloimmunization (e.g. immunoprophylaxis failure) when the recipient is in a state of viral-like inflammation. Prior studies suggested that type 1 IFN (IFN-α/β) generated in recipients after poly (I:C) treatment was sufficient but not necessary to lead to immunoprophylaxis failure (3), leading us to search for other pathways. The take home point of the current studies is that recipient complement is a key mediator in inflammation-associated immunoprophylaxis failure. As such, we propose a “two-hit” hypothesis, where the first “hit” is recipient inflammation, and the second “hit” is complement production/sensing (**Figure 7**).

Our findings thus support a model in which immunoprophylaxis fails in the setting of inflammation due to complement-mediated promotion of alloantibody generation. The classical complement pathway is activated by the binding of KELIg to KEL RBCs and the alternative complement pathway is activated by poly (I:C) (10–12). Free heme, generated as a result of RBC breakdown and known to activate the alternative complement pathway (13), may also be playing a role. Once generated, activated complement binds to complement receptors (CR1/2) on B-cells to lower the activation threshold for antibody generation. The requirement of C3 or C1q for immunoprophylaxis failure support this model. Further, the requirement of CR1/2 for immunoprophylaxis failure and the decreased binding of complement coated opsonized RBCs *in-vitro* to CR1/2^-/-^ B-cells lead us to suspect that B-cells play a critical “gatekeeper” role in our model.

CR1/2 on B-cells have been described to function as type 1 IFN (IFN-α/β) receptors (14), something of interest given our past studies showing that type 1 IFN is sufficient but not necessary for inflammation-associated immunoprophylaxis failure. It is likely that consumption of antibody coated RBCs by inflammatory monocytes, potentially driven by type 1 IFN production or the anaphylatoxins C3a and C5a [and presumably resulting in the generation of cytokines including type 1 IFN as well as C3a and C5a which are further known to activate antigen presenting cell/T-cell interactions and to create feedback loops (15, 16)], contribute in part to our findings. In addition to serving as type 1 IFN receptors, CR1/2 are key markers of marginal zone B cells that distinguish them from follicular B cells; past studies in the KEL system have shown the necessity of marginal zone B-cells but not follicular B-cells for anti-KEL IgG responses (17). Recent studies suggest that marginal zone B cell-mediated RBC alloimmunization may not require transport of RBCs to the splenic follicle (18), pointing to an intrinsic role of CR1/2 in B cell signaling following engagement of RBCs bearing both alloantigen and C3 split products. While prior studies demonstrated the importance of CR1/2 on B cells in anti-KEL IgG responses in the absence poly (I:C) (19), we cannot exclude the relevance of follicular dendritic cells to our present findings. Of note, models of systemic lupus erythematosus have shown the uptake of complement associated antigen by follicular DCs generates type 1 IFN in an IRF-5 dependent pathway (20).

Past studies have taught us that recipient complement and Fcγ receptors in combination are important in incompatible RBC clearance, KEL antigen modulation, and immunoprophylaxis efficacy when a recipient is in a baseline state of health (6). Although we previously thought RBC clearance patterns might impact immunoprophylaxis efficacy, recent studies (3) (including the present studies) show that may not be true in all settings. The data presented here support observations by Stegmann (21) and others (22) that RBC clearance patterns alone may be inadequate to fully evaluate the immunoprophylaxis efficacy of candidate monoclonal or polyclonal antibodies.

Our model and findings may have translational relevance for better understanding immunoprophylaxis failures (to RhIg). It remains unclear why some women become alloimmunized against the D antigen during pregnancy/childbirth despite prophylaxis with polyclonal RhIg (21, 23). FcγR 2/3 polymorphisms have been evaluated in such women, with the conclusion that high-affinity alleles encoding these receptors do not influence RhIg efficacy. Although antibodies to the RhD antigen are thought not thought to fix complement on RBCs themselves, it is possible that background complement activation or C3a/C5a generation as a result of heme or inflammation-associated complement pathway activation may play a role. Increasing evidence also supports the role of complement activation (24) in the pathophysiology of antibody positive as well as antibody negative (25) delayed hemolytic transfusion reactions, with complement blockade being shown to mitigate life-threatening reactions (26).

Study limitations should be considered. These studies were focused on a single blood group antigen and a single type of inflammation. Another consideration is that responses to different antigens on murine RBCs involve different pathways, with some being T-cell dependent (27) and others being T-cell independent; this is presumably true in humans as well. Complement has recently been shown to serve as a “switch” between CD4+ T-cell dependent and independent responses (19) and it is possible that poly (I:C) induced immunoprophylaxis failure is overcome by the CD4+ T-cell dependency of the alloantibody response in complement deficient mice. Our KELIg preparation is polyclonal with all IgG subtypes represented; monoclonal antibodies, either individually or in combination, may give different results (28). Further, in contrast to KEL, immunoprophylaxis to murine RBCs expressing an alternate (HOD, containing HEL) antigen can occur independent of complement or Fcγ receptors, suggesting that distinct antigen-antibody combinations may dictate the relative influence of complement, Fcγ receptors and inflammation on the success or failure of immunoprophylaxis (29–31). Indeed, anti-KEL and anti-HEL antibodies can induce antigen specific immunoprophylaxis following exposure to RBCs expressing both the KEL and HOD antigens (32). In contrast, antibodies directed toward one antigen may enhance or inhibit alloantibody formation against the non-target alloantigen (32–34). As such, distinct mechanisms of immunoprophylaxis should be considered depending on the target alloantigen involved (35, 36). One final consideration is that co-engagement of the B-cell receptor and complement receptors in humans may lead to different responses than those observed in mice (37).

In conclusion, our data highlight the critical role that complement plays in inflammation-induced immunoprophylaxis failure. Generated in one model, the mechanisms described here likely have broader applicability. Although complement was identified as contributing to disease more than half a century ago (38), it is likely that the era of studying and targeting complement activation and break down products, in transfusion medicine and beyond, has just begun.

## Data Availability Statement

The original contributions presented in the study are included in the article/**Supplementary Material**, further inquiries can be directed to the corresponding author.

## Ethics Statement

The animal study was reviewed and approved by Yale University IACUC.

## Author Contributions

VE-R, MS, JL, and JH planned the experiments. VE-R, MS, and JL executed most of the experiments. All authors made experimental suggestions and interpreted experimental results. JH wrote the initial draft of the manuscript, and all authors edited the manuscript and approved the final version of the manuscript.

## Funding

This work was funded by NIH/NHLBI (R01 HL126076 and R01 HL132951) to JH, 5 K08 HL141446 to DG, and P01 HL132819 to JZ. It was also funded in part by NCI P30CA016359 and NIH/NIDDK U54 DK106857 to Yale University.

## Conflict of Interest

The authors declare that the research was conducted in the absence of any commercial or financial relationships that could be construed as a potential conflict of interest.
