# Supplementary material for: Complement Plays a Critical Role in Inflammation-Induced Immunoprophylaxis Failure in Mice
Source: Front Immunol. 2021 Jun 25;12:704072. doi: 10.3389/fimmu.2021.704072 (PMC8270673; doi:10.3389/fimmu.2021.704072)

**Complement plays a critical role in inflammation-induced immunoprophylaxis failure in mice**

Vicente Escamilla-Rivera^1^, Manjula Santhanakrishnan^1^, Jingchun Liu^1^, David R. Gibb^2^, James E. Forsmo^1,3^, Ellen F. Foxman^1,4^, Stephanie C. Eisenbarth^1,4^, C. John Luckey^5^, James C. Zimring^5^, Krystalyn E. Hudson^6^, Sean R. Stowell^7,8^, and Jeanne E. Hendrickson^1,9^

1. Yale University School of Medicine, Department of Laboratory Medicine, New Haven, CT
2. Cedars-Sinai Medical Center, Department of Pathology & Laboratory Medicine, Los Angeles, CA
3. Georgia Institute of Technology, Department of Biomedical Engineering, Atlanta, GA
4. Yale University School of Medicine, Department of Immunobiology, New Haven, CT
5. University of Virginia, Department of Pathology, Charlottesville, VA
6. Columbia University Irving Medical Center, Department of Pathology and Cell Biology, New York, NY
7. Harvard Medical School, Joint Program in Transfusion Medicine, Brigham and Women’s Hospital, Department of Pathology, Boston, MA
8. Harvard Medical School, Harvard Glycomics Center, Beth Israel Deaconess Medical Center, Boston, MA
9. Yale University School of Medicine, Department of Pediatrics, New Haven, CT

Corresponding author: Jeanne E. Hendrickson, MD; Yale University Departments of Laboratory Medicine and Pediatrics, 330 Cedar Street, Clinic Building 405, PO Box 208035, New Haven, CT 06520-8035; email: jeanne.hendrickson@yale.edu; phone: (203) 737-8316; fax: (203) 785-5029

**Supplemental Figure 1. KEL RBC clearance patterns after KELIg, in the absence of poly(I:C).**  KEL RBCs were labeled with DiO and mixed with wild type RBCs labeled with DiI; this mixture was transfused into recipients that had been infused with KELIg. (**A**) Wild type and C3^-/-^ recipients are shown; (**B**) Wild type and C1q^-/-^ recipients are shown.

**A.**


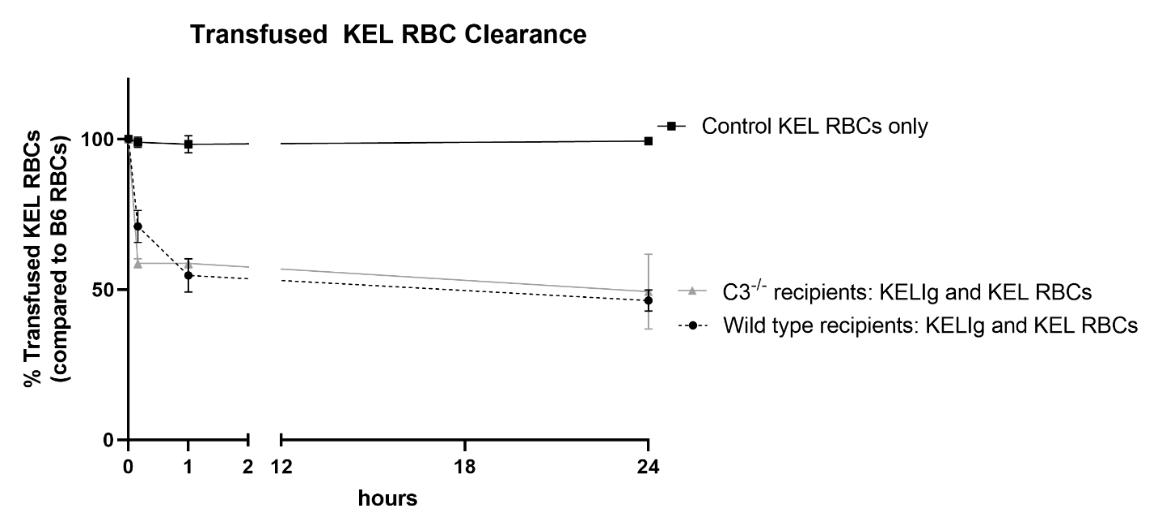


**B.**

**Supplemental Figure 2. Gating strategy for splenocytes**. After gating on single cells, live cells, and non-T-cells/non-B-cells/non-RBCs, splenic red pulp macrophages, dendritic cells (CD8α and CD11b and plasmacytoid), resident and inflammatory monocytes, and neutrophils were evaluated.


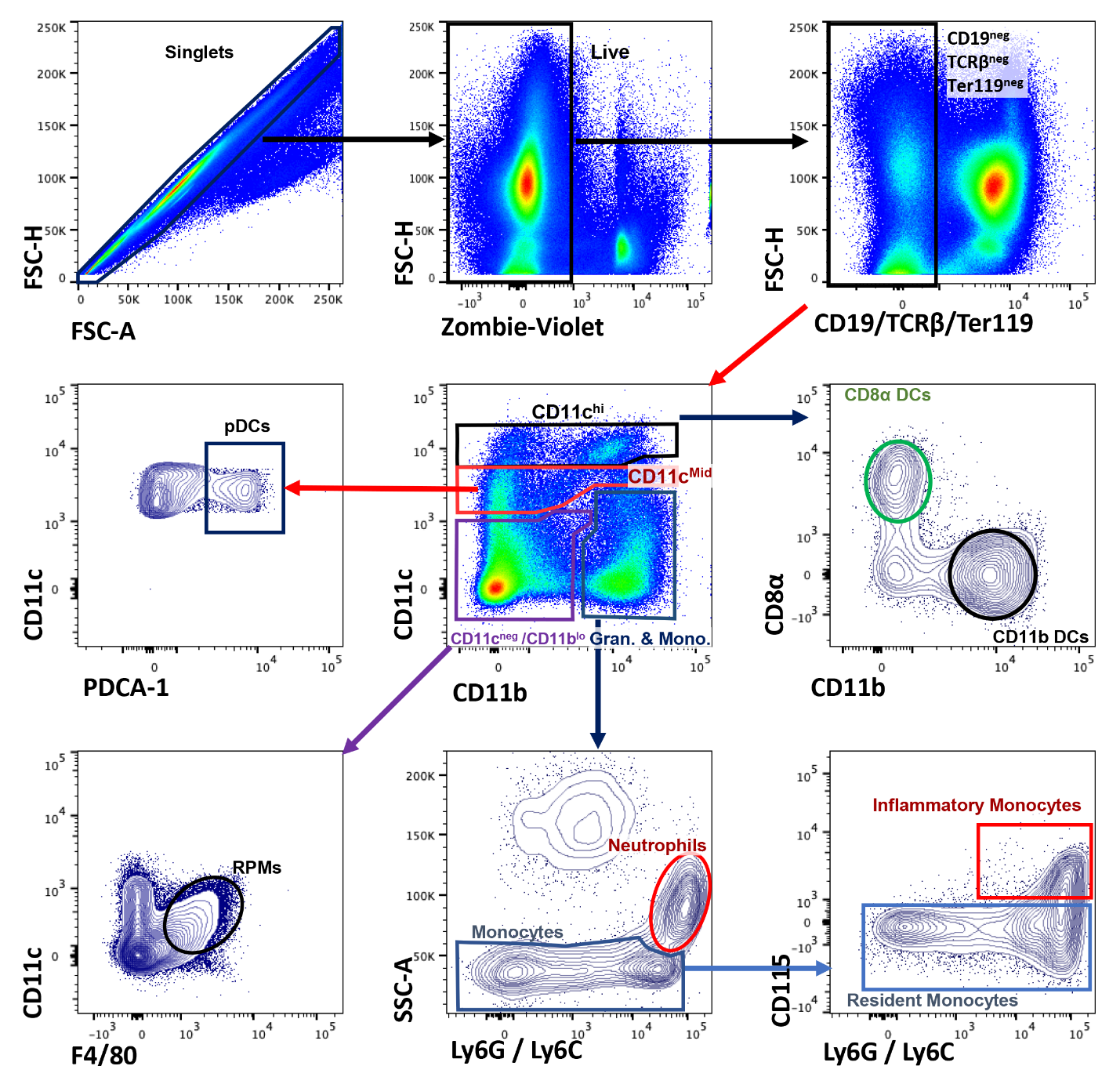


**Supplemental Figure 3**. **Poly (I:C) and KELIg immunoprophylaxis result in increased transfused KEL RBC consumption by peripheral blood inflammatory monocytes in wild type compared with C3^-/-^ mice.** DiO labeled KEL RBCs were transfused to wild type or C3^-/-^ mice treated with KELIg in the presence or absence of poly (I:C), and the percent of non-T/non-RBC peripheral blood cells were evaluated at one hour (**A**) and 16 hours (**B**) post-transfusion. These cell subsets were then evaluated for DiO fluorescence patterns, after first excluding TER119 positive RBCs on the surface of the WBCs; **(C)** shows the one hour and (**D**) shows the 16-hour time point. These data are representative of 2 independent experiments with 3 mice/group/experiment; error bars indicate standard deviation between mice.



**Supplemental Figure 4**. **Negative control conditions for *in vitro* B-cell assays.**  DiO labeled KEL or wild type RBCs were incubated with KELIg (or not) in the presence (or absence) of sera, followed by incubation with peripheral blood derived WBCs from wild type donor mice. (**A**) In wells using *wild type* RBCs, KELIg, sera, and WBCs from wild type donor mice, B220+ B-cells cells were separated by DiO positivity; the DiO positive and negative populations were then evaluated for their C3 positivity. Shaded histograms are the DiO positive population, open histograms are the DiO negative population. (**B**) In wells using KEL RBCs, *no KELIg*, sera, and WBCs from wild type donor mice, the same gating was completed. (**C**) In wells using KEL RBCs, KELIg, *no sera*, and WBCs from wild type donor mice, the same gating was completed. These data are representative of 3 independent experiments.


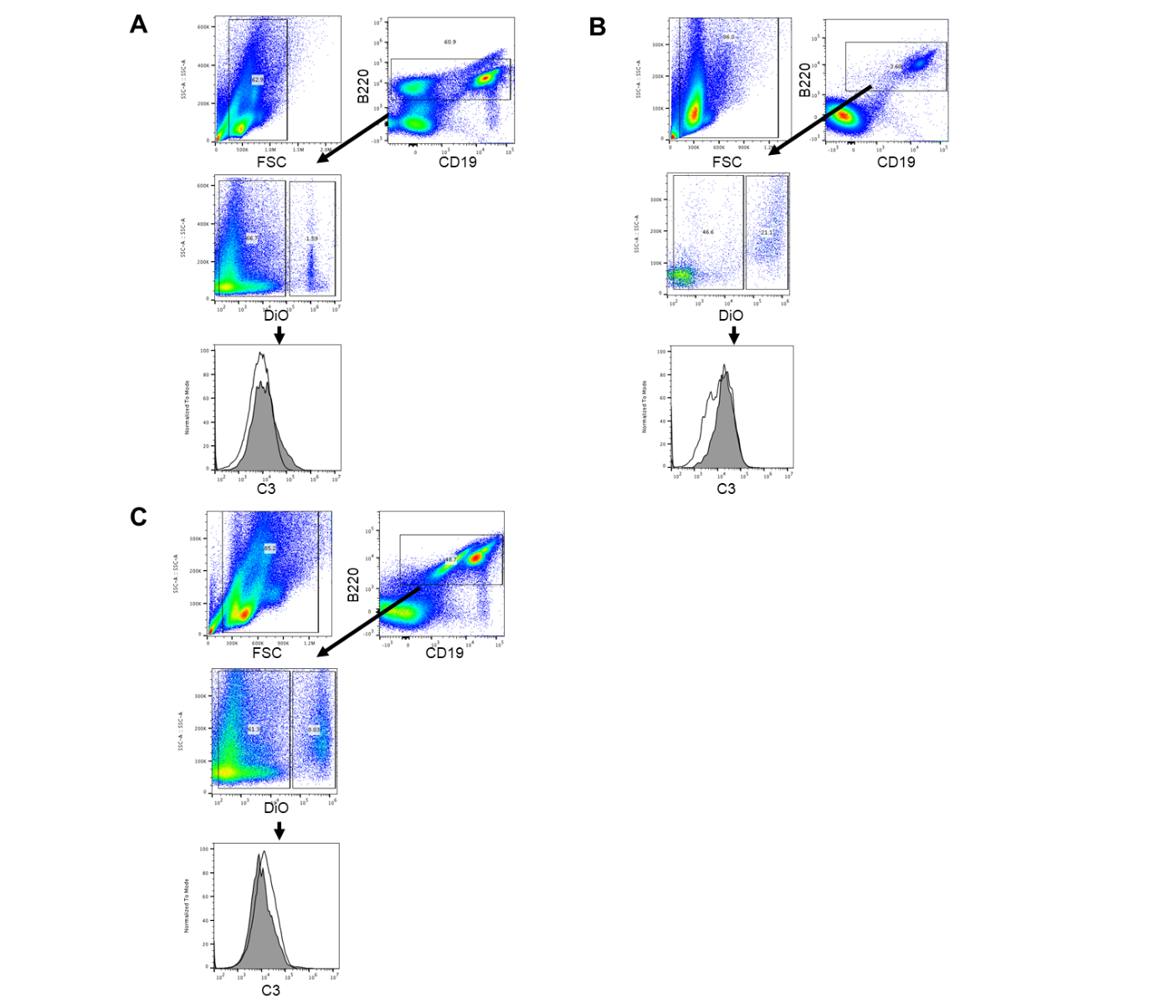

Supplement: Supplementary file 1 [file DataSheet_1.docx]
